# Workplace Violence and Burnout among Health Workers Two Years after the COVID-19 Outbreak in Wuhan, China: The Chain Mediation Effect of Sleep Disturbance and Work Ability

**DOI:** 10.3390/healthcare12181903

**Published:** 2024-09-23

**Authors:** Fuyuan Wang, Min Zhang, Nian Xiong, Yiming Huang, Yuting Tang, Chuning He, Xinxin Fang, Xi Fang, Lan Chen, Qing Zhang

**Affiliations:** 1 School of Population Medicine and Public Health, Chinese Academy of Medical Sciences & Peking Union Medical College, Beijing 100730, China; wangfuyuan@student.pumc.edu.cn (F.W.); huangyiming@sph.pumc.edu.cn (Y.H.); tyting0109@student.pumc.edu.cn (Y.T.); hechuning@student.pumc.edu.cn (C.H.); s2023027004@pumc.edu.cn (X.F.); 2 Department of Neurology, Union Hospital, Tongji Medical College, Huazhong University of Science and Technology, 1227 Jiefang Ave, Wuhan 430022, China; nianxiong@hust.edu.cn; 3 Wuhan Red Cross Hospital, Wuhan 430000, China

**Keywords:** COVID-19, burnout, workplace violence, sleep disturbance, work ability, mediation analysis, health workers

## Abstract

Background: Health workers (HWs) faced considerable psychosocial hazards during the COVID-19 pandemic, which profoundly affected their occupational health and job performance. The potential indirect relationship between workplace violence (WPV) and burnout among HWs needs to be further explored. The purpose of this study is to examine the chain mediating effects of sleep disturbance and work ability in the relationship between WPV and burnout among HWs. Methods: A cross-sectional study was conducted in a secondary hospital two years after the COVID-19 outbreak in Wuhan, China. A sample of 571 HWs was recruited using a cluster sampling method, achieving a response rate of 80.06%. Participants completed self-report questionnaires that included demographic information and measures of WPV, burnout, sleep disturbance, and work ability. Results: The prevalence rates of burnout and WPV among HWs two years after the COVID-19 outbreak were 37.30% and 31.52%, respectively. WPV was significantly associated with burnout (β = 0.446, *p* < 0.001). Sleep disturbance was identified as a mediator in the relationship between WPV and burnout (β = 0.063, 95% CI: 0.027–0.105), accounting for 14.13% of the total effect. Similarly, work ability also played a mediating role in this relationship (β = 0.142, 95% CI: 0.065–0.225), accounting for 31.84%. Additionally, both sleep disturbance and work ability exhibited a chain mediation effect on the association between WPV and burnout (β = 0.020, 95% CI: 0.008–0.036), and the total indirect effect accounted for 50.67%. Conclusions: Among Chinese HWs, WPV exerts significant direct and indirect effects on burnout symptoms, mediated by sleep disturbance and work ability. This finding provides valuable empirical insights for designing interventions to mitigate the adverse effects of psychosocial factors such as WPV and burnout among HWs. After exposure to WPV, measures focused on reducing sleep disturbance and enhancing work ability may prove effective in alleviating burnout in subsequent interventions.

## 1. Introduction

Ensuring occupational safety and health (OSH) in the workplace is foundational for advancing the “Decent work for all” policy advocated by the Sustainable Development Goals (SDGs) of the United Nations [1]. Health workers (HWs) are particularly susceptible to significant psychosocial hazards in healthcare settings, including burnout, work-related stress, sleep disturbances, and various forms of workplace violence (WPV). Particularly during the COVID-19 pandemic, HWs were at an increased risk of experiencing psychological consequences compared to the general population, which profoundly impacted their well-being and work performance [2]. To mitigate the potential consequences of psychosocial factors, proactive measures are increasingly implemented across numerous workplaces. Endorsed by the SOLVE (Integrating Health Promotion into Workplace OSH Policies) training program [3], a tool of the International Labour Organization (ILO) designed to address workplace psychosocial hazards and risks, health promotion programs at the workplace can be developed to enable workers to cope with psychosocial factors more effectively, such as stress, violence, or unhealthy sleep. Additionally, integrating health promotion into OSH policies not only enhances occupational health practice but also fosters a culture of prevention. Thus, it plays a pivotal role in elucidating the interplay between these psychosocial hazards.

Burnout is a psychological syndrome characterized by job-related stress in various healthcare practice environments [4]. It comprises the following three dimensions: emotional exhaustion, depersonalization, and a diminished sense of personal accomplishment [5]. The COVID-19 pandemic introduced a multitude of psychological stressors, leading to numerous reports of unprecedented levels of burnout [6]. Additionally, the prevalence of burnout was rampant, reaching global levels in occupational settings, especially among professional HWs [7,8,9]. Studies have demonstrated that the prevalence of burnout among physicians was 82.1% in mainland China [10], 38.2% in the United States [11], and 2.5% to 87.9% in low- and middle-income countries as reported from a meta-analysis across 43 studies [12]. Recently, burnout has been classified as an occupational phenomenon rather than a medical condition in the 11th Revision of the International Classification of Diseases (ICD-11) by the World Health Organization (WHO) [13]. It exerts adverse effects on HWs and the healthcare system, manifesting in poor physical and mental health, impaired work–life balance, and reduced quality of patient care [7,14,15]. Additionally, burnout can escalate both direct and indirect medical costs [15,16].

WPV is conceptualized by the ILO as “a range of unacceptable behaviors and practices, or threats thereof, whether a single occurrence or repeated, that aims at, result in, or are likely to result in physical, psychological, sexual or economic harm, and includes gender-based violence and harassment” [17]. Characterized by its nature of intolerable and resulting harm, WPV is prevalent across diverse work environments and occupational groups. On a global scale, the COVID-19 pandemic has been associated with exacerbated incidences of WPV against HWs, even considering the variability of the data [18,19]. Hadavi demonstrated that the overall prevalence of WPV among HWs during the COVID-19 pandemic was 45.80% [20], with psychiatric nurses being the most affected professionals [21]. Zhang et al. reported HWs employed during the COVID-19 pandemic faced a higher risk of both physical and psychological WPV [22]. According to another study focusing on the gender disparity among HWs, it showed that the prevalence of WPV tended to be higher toward men in Asia, while it was higher toward women in Latin America during the COVID-19 pandemic [23]. WPV poses a serious threat to the safety and well-being of HWs in multiple dimensions, leading to diminished job satisfaction, increased occupational burnout, and severe psychological issues, including post-traumatic stress disorder and suicidal ideation [24,25]. Data also show the possible consequences in physicians’ burnout and the deterioration of quality of life, which are linked to physical and mental health problems, which, in a domino effect, fall on patients’ quality of care [26,27]. Additionally, it presents a significant obstacle to achieving the objective of the “Decent work for all” policy of the SDGs [1].

Recent research has consistently shown a correlation between exposure to WPV and heightened levels of burnout. Notably, a substantial proportion of the participants across these studies were HWs [28,29,30]. Some other studies have reported the indirect relationship with WPV predicting burnout, showing that there may be different mediators between them, such as work ability, a sense of coherence, and depression [31,32,33], while research during the COVID-19 pandemic remains limited. Therefore, to contribute insights to the development of integrated health promotion policies for OSH, further investigation into potential mediating factors between WPV and burnout during and after the COVID-19 pandemic is needed.

Sleep disturbance, as an occupational hazard, is prevalent in almost four out of ten HWs [34]. Strong evidence indicates that insufficient sleep is associated with substantial health and cognitive performance consequences [35,36]. At the same time, work ability also served as a comprehensive concept for occupational health and prevention. For example, Shechter et al. conducted a cross-sectional study and highlighted that poor sleep and insomnia symptoms were significantly associated with the emotional exhaustion aspect of burnout [37]. Converso et al. have explored whether work ability affects the burnout of HWs [38,39]. Additionally, previous research has demonstrated that exposure to WPV can increase the risk of sleep disturbance and diminished work ability [40,41,42]. However, there have been limited investigations on the interplay and relationship between WPV, sleep disturbance, work ability, and burnout [43,44].

Given the constraints of the previous studies, including the potential indirect relationship between WPV and burnout during and after the COVID-19 pandemic, as well as the limited investigations into the interplay between WPV, sleep disturbance, work ability, and burnout, this study aims to investigate the relationship between WPV, sleep disturbance, work ability, and burnout among HWs two years after the COVID-19 outbreak. The findings will address pressing implications for health promotion and psychosocial risk factors for occupational safety and health (OSH). The study will also address the urgent implications for health promotion related to psychosocial risk factors for OSH. To address this objective, we formulated the following hypotheses: Firstly, there exists an association between WPV and burnout among HWs in China. Secondly, sleep disturbance and work ability mediate the relationship between WPV and burnout, respectively. Lastly, sleep disturbance and work ability exhibit a chain mediation effect between WPV and burnout.

## 2. Materials and Methods

### 2.1. Study Design and Participants

This cross-sectional study was conducted at a secondary general hospital (hereafter referred to as “the sample hospital”) between June and July 2022 in Wuhan, Hubei Province, China. This hospital is located merely a mile away from the Wuhan Huanan Seafood Wholesale Market, and it was among the first hospitals designated for COVID-19 infections in Wuhan between 21 January and 6 March 2020. It has 477 open beds and 27 clinical specialties, including intensive care units, the most famous of which are the Sleep Medicine Center, Thyroid and Breast Surgery, Endocrinology, Pain Medicine, Neurology, and Obstetrics. In 2021 and 2022, the ILO-Peking Union Medical College (ILO-PUMC) team conducted both online and in-person Work Improvement in Health Services (HealthWISE) training workshops at this hospital. The workshop consisted of the following four sessions: thematic reports on national and international OSH, good practices from HealthWISE pilot hospitals, instruction on HealthWISE modules, and walk-through interventions in this hospital. A total of 23 department heads and hospital leaders from this hospital participated in the training workshop and demonstrated significant improvements in knowledge awareness, beliefs and attitudes, and capacity building after the training. The effectiveness of this workshop highlighted its crucial role in assisting this hospital in establishing a fundamental framework for OSH management systems specifically tailored to HWs.

Among hospitals nationwide, we considered the sample hospital as a cluster and included all of its HWs who met our criteria as participants. A sample of 574 HWs was recruited through the cluster sampling method between June and July 2022, achieving a response rate of 80.06%. The sample covered all departments within the hospital. Inclusion criteria included the following: (a) certification as a healthcare professional; (b) voluntary participation with informed consent; (c) employment at the hospital as a regular employee for over one year. Exclusion criteria included the following: (a) absence from duty for over one month during the investigation period; (b) failure to complete the questionnaire in the opening hours.

An online version of the questionnaire was distributed to entire staff of the hospital through Wenjuanxing, a widely used web-based survey platform in China. A total of 574 questionnaires were collected, with 3 excluded due to logical errors (such as an age less than 5 years old or inconsistencies between work years, title, and salary) or missing key values (such as WPV, burnout, WAI, or sleep), resulting in a final sample of 571 questionnaires.

### 2.2. Ethics Statement

This study was conducted after approval from the Chinese Academy of Medical Sciences & Peking Union Medical College Ethics Committee (Registration number CAMS&PUMC-IEC-2022-044) in 2022. Participation was voluntary and informed consent was presented on the first page of the questionnaire. All participants were provided with detailed explanations regarding the attributes, benefits, uses, and potential adverse effects of the study. All data were anonymized to ensure the privacy and confidentiality of the participants. Additionally, participants were assured that their data would be used solely for research purposes.

### 2.3. Measurement of Burnout

Burnout was assessed using the Chinese version of 2 single-item measures adapted from the full Maslach Burnout Inventory (MBI) [5]: (1) “I felt burned out from my work”; (2) “I have become more callous toward people since I took this job”. As assessed by the full MBI, these 2 items are strongly associated with the emotional exhaustion and depersonalization domains of burnout, with the area under the receiver operating characteristic curve of 0.94 for the single item of emotional exhaustion and 0.93 for the single item of depersonalization [45,46]. The 2-item scale has been widely adopted by numerous scholars in previous research to assess burnout among HWs [11,47]. We used a 7-point Likert scale ranging from 0 (never) to 6 (every day). The total score ranged from 0 to 12, with higher scores indicating more severe levels of burnout. Individuals with a high score (frequency≥ once per week/score ≥ 4) on either of these two items were identified as having burnout symptoms [45,46]. 

### 2.4. Measurement of WPV

The Chinese version of Workplace Violence in the Health Sector Country Case Studies Research Instruments Survey Questionnaire [48] was used to assess WPV among HWs. Developed by the ILO and the WHO, this questionnaire was translated into Chinese by Liu et al. [49,50]. This scale is widely utilized in China and has previously been employed to evaluate WPV among Chinese HWs [51,52,53,54]. The Cronbach’s α coefficient for this scale was 0.83 [49]. It categorizes WPV into several types, including physical violence and psychological violence, which encompass verbal abuse, bullying/mobbing, racial harassment, and sexual harassment. The complete questionnaire was detailed in our prior research [49,55]. In this study, WPV was evaluated based on the frequency of incidents reported by HWs in the year before the investigation day. Response options included the following: 0 (never), 1 (1 time), 2 (2–4 times), 3 (5–10 times), 4 (several times per month), and 5 (almost every day). The total score ranged from 0 to 25, with higher scores indicating more severe levels of WPV experienced by HWs. Individuals scoring equal to or higher than 1 (frequency ≥1 time) on any type of WPV were classified as having experienced WPV. 

### 2.5. Measurement of Work Ability

The work ability of HWs was measured using the Chinese version of the Work Ability Index (WAI), which was translated by Zhang et al. in 2003 [56,57]. The WAI serves as a universal instrument for evaluating self-assessed work ability concerning job demands, health status, and personal resources [58]. It has been widely validated [59,60] and extensively utilized [38,61]. In the present study, Cronbach’s α coefficient was 0.78. It comprised the following 7 sections: (1) current work ability compared with lifetime best (score range: 1–10), (2) work ability in relation to mental and physical demands (score range: 2–10), (3) number of diagnosed diseases (score range: 1–7), (4) work impairment due to diseases (score range: 1–6), (5) sick leave over the past 12 months (score range: 1–5), (6) self-prognosis of work ability in the next 2 years (score range: 1–4 or 7), and (7) mental resources (score range: 1–4). The total score ranged from 7 to 49 and was categorized into the following four levels: excellent (44–49), good (37–43), moderate (28–36), and poor (7–27). 

### 2.6. Measurement of Sleep Disturbance

Sleep disturbance was assessed through self-reported responses on the following three questions, with options for “yes” or “no” answers: (1) “Did you experience difficulties falling asleep at night?” (2) “Did you frequently awaken in the early hours, unable to return to sleep?” (3) “Did you consume sleeping pills more than three times per week?” These questions have been widely used in epidemiological studies to evaluate sleep disturbance in adults [62,63,64]. In this study, the cumulative number of affirmative responses (ranging from 0 to 3) was considered as the mediating variable.

### 2.7. Statistical Analyses

All data analysis and processing were conducted using the BruceR packages (https://psychbruce.github.io/bruceR/, accessed on 10 March 2024) in R software version 4.3.2 (R Development Core Team, Vienna, Austria). Firstly, descriptive analysis, the independent-samples *t*-test, and one-way analysis of variance were used to describe and compare the demographic characteristics of the study population. Secondly, Spearman’s correlation analysis was used to investigate the relationships between burnout, WPV, sleep disturbance, and work ability. Finally, to assess the potential chain mediation effects, we utilized a bootstrap method using bias-corrected 95% confidence intervals (BC 95% CIs). It was estimated through the Serial-Multiple Mediation Model 6 of PROCESS macro version 3.5 in R, developed by Hayes [65]. Hayes argued that bootstrapping is the most powerful method to assess indirect effects because it does not rely on assumptions regarding the sampling distribution of the indirect effect and offers better control over type I errors [66]. The indirect effect and BC 95% CIs were based on 10,000 bootstrapping samples. If the BC 95% CIs did not contain zero, the mediating effect was considered significant. Gender, age, department, occupation, years of working, number of employees coworking, shift work, night work, and direct contact with patients were treated as covariates. Harman’s univariate test, conducted using SPSS 25, assessed the severity of the common method bias [67].

## 3. Results

### 3.1. Participant Characteristics and Prevalence of WPV and Burnout

The results of Harman’s univariate test revealed five factors with a characteristic root >1. Among these, the first factor accounted for 24.71% of the variance, which is below the critical standard of 40.00%. Consequently, the findings suggest that there is no significant common method bias in this study.

Table 1 presents a summary of the demographic characteristics of the participants and the difference in burnout scores between groups. A total of 571 valid questionnaires were collected in our study. Participant ages ranged from 19 to 69 years old, with a mean of 37.11 ± 8.53 years old (mean ± SD). Female HWs comprised 78.46% of the sample. Table 1 illustrates the significant differences in the burnout scores among HWs in age, occupation, number of employees coworking, shift work, night work, and direct contact with patients. Considering the results of our previous studies and the differences in burnout shown in Table 1, we considered the above variables as confounders and adjusted them in the chain mediation analysis.

**Figure 1:**
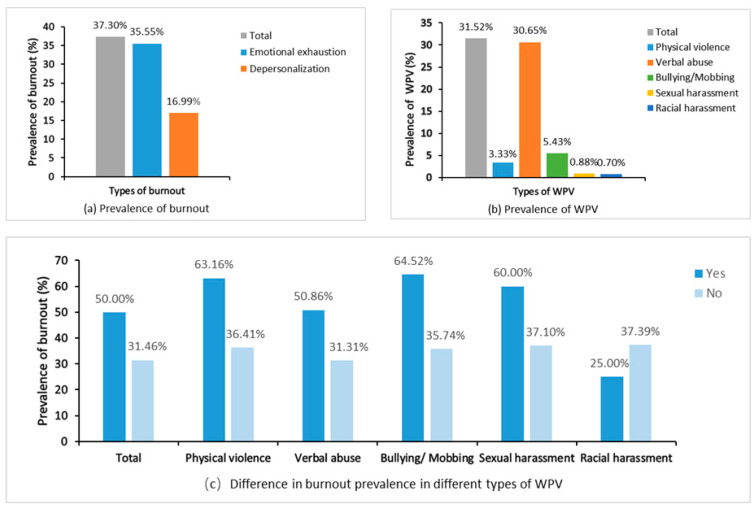
Prevalence of burnout and WPV among HWs two years after the COVID-19 outbreak. Note: WPV, workplace violence.

Among all 571 participants, the mean scores of burnout and WPV were 5.07 ± 3.13 and 0.87 ± 1.66, respectively. Two years after the COVID-19 outbreak, a total of 180 (31.52%) and 213 (37.30%) HWs fulfilled the criteria for WPV and burnout, respectively (Figure 1a,b). When comparing participants with and without an experience of WPV two years after the COVID-19 pandemic, those meeting the criteria for any type of WPV, except racial harassment, reported a higher prevalence of burnout (Figure 1c).

### 3.2. Correlation Analysis

Table 2 reveals that burnout exhibited a positive correlation with WPV (r = 0.256, *p* < 0.001) and sleep disturbance (r = 0.322, *p* < 0.001), while demonstrating a negative correlation with work ability (r = −0.454, *p* < 0.001). The *p* values associated with these correlations were statistically significant. The correlation coefficient indicated effect sizes exceeding 0.2 between the variables.

### 3.3. The Chain Mediating Effect of WPV on Burnout

**Figure 2:**
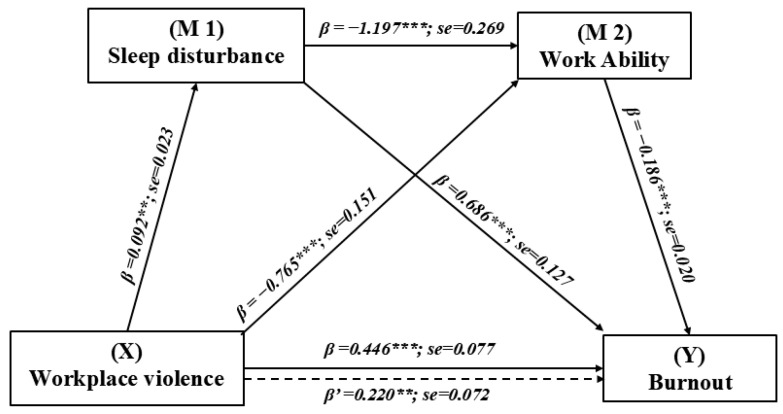
Chain mediation effect of sleep disturbance and work ability in the relationship between WPV and burnout with non-standardized beta values and standard error. X is the predictor, M1 and M2 are mediators, and Y is the outcome variable. ** *p* < 0.01; *** *p* < 0.001.

The indirect and specific effects of WPV on burnout, mediated through sleep disturbance and work ability, is shown in Table 3. Figure 2 shows the chain mediation model. After adjusting for covariates, the total effect of WPV on burnout was found to be significant (c = 0.446, SE = 0.077, *p* < 0.001). When accounting for all variables (including covariates) in the model, the pathways through the single mediation of sleep disturbance (β = 0.063; bias-corrected (BC) 95% CIs: 0.027, 0.105), single mediation of work ability (β = 0.142; BC 95% CIs: 0.065, 0.225), and both mediators (β = 0.020; BC 95% CIs: 0.008, 0.036) were all statistically significant. These pathways accounted for 14.13%, 31.84%, and 4.48% of the total effect, respectively. The overall indirect effect, as measured through the mediation of sleep disturbance and work ability, was found to be statistically significant (β = 0.226; BC 95% CIs: 0.133, 0.321). This indirect effect accounted for 50.67% of the total effect size, indicating that the pathway through both mediators plays a significant role in the relationship between WPV and burnout. Additionally, the indirect effect through both sleep disturbance alone and work ability alone was also significant.

This study analyzed divergent outcomes to determine if specific indirect effects mediated by certain factors were stronger than others. As indicated in Table 3, all three contrasts demonstrated statistically significant results, with the BC 95% CIs excluding the zero-point estimate. According to the comparison of these specific direct effects, we observed that both pathways mediated solely by sleep disturbance and solely by work ability exhibited stronger mediating effects compared to the pathway mediated through a chain of sleep disturbance and work ability.

## 4. Discussion

To the best of our knowledge, this investigation represents the first to examine the chain mediation relationships involving sleep disturbance and work ability in the association between WPV and burnout among HWs in China. Overall, we revealed the prevalence of burnout and WPV among HWs two years after the COVID-19 outbreak, which were 37.30% and 31.52%, respectively. Consistent with our hypotheses, the result demonstrates a positive association between WPV and burnout in HWs. Moreover, regarding the hypothesis of the mediating relationship, the positive predictive effect of WPV on burnout is mitigated by the buffering influence of sleep disturbance and work ability. Therefore, our research findings validate all of the hypotheses posited.

### 4.1. Prevalence of Burnout and WPV Two Years after the COVID-19 Outbreak

This study revealed a notable prevalence rate of WPV (31.52%) and overall burnout (37.30%) among HWs two years after the COVID-19 outbreak, as shown in emotional exhaustion (35.55%) and depersonalization (16.99%). A meta-analysis encompassing 239 studies showed that the burnout prevalence rate among HWs exposed to COVID-19 was 37% [68], slightly lower than the 37.30% of our study. However, a similar cross-sectional study conducted in six Wuhan hospitals two years after the pandemic revealed a comparatively higher burnout rate (67.09%) than that identified in our study [69], which may reflect the differences in the impact of outbreaks in different regions. Compared to the beginning of the epidemic, this study shows that the prevalence of burnout is still higher, suggesting that the impact of the epidemic on HWs’ mental health is long-term. This is also supported by the study by Hu et al. [70] and the systematic review by Macaron et al. [71], where the prevalence of burnout remained significant even in the later stages of the epidemic. A systematic review by Macaron et al. showed the prevalence of burnout among physicians during the early pandemic period was 60.7% compared to 49.3% during the late pandemic period [71]. The COVID-19 pandemic has led to persistently high levels of burnout and WPV among healthcare professionals [70].

Our survey revealed similar results in terms of the prevalence of WPV. A comprehensive meta-analysis has reported that the global prevalence of any form of WPV during the COVID-19 epidemic was 43% among HWs. This figure is notably higher than the 31.52% prevalence rate observed in our study. This variance could potentially be attributed to the conduction of the HealthWISE training at the sample hospital by the ILO-PUMC team in both 2020 and 2021, which highlights the importance of targeted training and interventions in reducing WPV. Before conducting this survey, the sample hospital had received certain occupational health interventions and guidance on burnout and WPV, potentially contributing to the maintenance of a moderate level of burnout. Although the prevalence of WPV in the sample hospitals was lower than the global average, more than one-third of HWs reported experiencing WPV, indicating that there is still significant potential to further reducing WPV and burnout levels. Consequently, hospital management needs to continue to pay attention and take measures to improve the working environment.

### 4.2. The Direct Effect of WPV on Burnout

The results of our study indicate a significantly positive correlation between WPV and burnout among Chinese HWs (r = 0.256, *p* < 0.001), which is in agreement with previous research [28,43]. Additionally, a survey conducted after a year of the COVID-19 pandemic revealed that exposure to WPV at “intermediate and high” levels is associated with a markedly higher risk of burnout among Bangladeshi nurses (RR = 3.65, 95% CI: 2.40–5.56) [72]. Some studies suggest that WPV may serve as a predictor of burnout, albeit indirectly. While experiencing verbal or physical violence in the workplace may not directly cause burnout, it can contribute through other factors, such as work ability, emotional reaction, and sense of coherence [31,32,38], ultimately resulting in significant burnout levels. This indirect pathway implies that interventions should target not only reducing WPV but also enhancing protective factors that mitigate the impact of WPV on mental health. The comparison of these studies calls for further research to explore the specific mechanisms through which WPV contributes to burnout and to evaluate the effectiveness of various intervention strategies.

As advocated by the SOLVE training program of the ILO, health promotion and prevention measures targeting psychosocial factors should be integrated into the risk management framework of a comprehensive OSH management system [3]. To mitigate the adverse effects of psychosocial factors such as WPV and burnout, the assessment and control of these factors should be regarded as essential practices. Therefore, identifying the intertwined correlation between WPV and burnout, including their direct and indirect pathways to other psychosocial factors, is crucial for effectively preventing adverse outcomes. Furthermore, it emphasizes the importance of translating research findings into practical actions that can be implemented in healthcare settings to protect the mental health of HWs.

### 4.3. The Mediation Effect of Sleep Disturbance and Work Ability

A novel finding in this study is the mediating effect of sleep disturbance between WPV and burnout. This result indicates that HWs who experience WPV are at an increased risk for sleep disturbance, subsequently leading to an increased level of burnout within their professional environment. Previous studies have consistently shown that sleep-related impairment was associated with various psychosocial outcomes [73], including increased burnout [37]. Sleep is an important process for physical and psychological recovery, and sleep deprivation can interfere with the restoration of physical and mental energy and resources [74]. In fact, insomnia or sleep disturbance often persists throughout a health worker’s entire career, especially during an international public health emergency such as the COVID-19 pandemic. A study conducted among the general population, HWs, and quarantined individuals during the COVID-19 pandemic reveals that the prevalence of sleep disturbances was the highest among HWs, at 29.8% [75]. Therefore, when HWs are exposed to violence, whether at the physical or psychological level, the consequences may initially manifest in their daily lives, such as shortened sleep duration and poor sleep quality, and subsequently lead to emotional exhaustion or depersonalization at work. From a preventive perspective, sleep interventions for HWs exposed to WPV should be implemented as follows: (1) disseminate information and guidance on issues related to healthy sleep; (2) ensure that shift and night work scheduling is fair, reasonable, and gender-sensitive; (3) provide targeted training on sleep and rest management for HWs engaged in night or shift work; (4) offer adequate support, counseling, and psychotherapy, as appropriate [3].

In addition, the results indicated that HWs who experienced WPV were more likely to experience declined work ability, subsequently causing an elevated level of burnout. Limited studies have explored the relationship between exposure to violence, work ability, and burnout. Our finding aligns with Converso’s research [38], which reported a similar mediating effect of work ability between WPV and burnout among nursing staff [76]. However, other studies have shown that work ability declines as burnout levels rise [77,78], which contrasts with the findings of this study. One possible explanation could be that work ability and burnout interact with each other and are mutually causal. People with strong work ability may be more capable of recovering from burnout, thereby mitigates its effects, while people with weaker work ability may find it more challenging to recover from burnout, which reflects the difference in resilience. Therefore, both of the above pathways between burnout and work ability could be reasonable [79]. In addition, workforce aging affects work ability differently. Hatch et al. [78] indicated that increased age correlates with declines in physical work ability. In contrast, they found that psychological work ability decreases with age only at higher burnout levels, while, at lower levels, it improves. Sottimano’s research underscores work ability as a mediator in the relationship between aging and emotional exhaustion [76]. Working ability is closely linked to overall health, and for HWs exposed to WPV, the recovery of work ability depends on a combination of professional knowledge as well as organizational, ergonomic, and psychosocial conditions in the workplace [80]. For hospitals and managers, it is crucial to promote HWs’ work ability through the following measures: (1) streamlining work tasks to be more autonomous; (2) improving work postures, work tools, and the workplace temperature according to work demands and environment; (3) optimizing work community, role clarity, and work experience in work organization; (4) expanding more possibilities for development and training for professional competence; (5) encouraging weight control, physical activities, and engagement in hobbies to support health and functional capacity [81,82].

### 4.4. The Chain Mediation Effect

To the best of our knowledge, the current study contributes to evaluating the chain mediating effect of sleep disturbance and work ability between WPV and burnout among HWs, a relationship that has not been explored previously. The findings of this study demonstrate that exposure to WPV is sequentially associated with an increased level of sleep disturbance initially, followed by decreased work ability, which subsequently exacerbates burnout. According to the SOLVE program of the ILO [3], this association suggests that integrated health promotion intervention targeting any steps may help mitigate the occurrence of adverse outcomes.

The results of the chain mediating analysis revealed that the total indirect effect of sleep disturbance and work ability accounted for 50.67% of the total effect size (B = 0.446, *p* < 0.001) between WPV and burnout. This percentage is comparable to that observed in other studies [31,38]. Yeping Fei et al. reported an indirect effect of 62.45% between WPV and burnout mediated by the sense of coherence [31] during the COVID-19 pandemic, surpassing 50.67% in our study. However, their investigation exclusively focused on nurses, which may not fully capture the experiences of all HWs.

Sleep disturbance and work ability are key factors in mitigating burnout. Our study provides further clarity by demonstrating that decreased sleep disturbance levels and improved work ability could be beneficial for HWs in alleviating burnout symptoms after exposure to WPV. The mediating mechanisms involving sleep disturbance and work ability provide valuable insights into addressing the occupational health challenges encountered by HWs after the COVID-19 outbreak. In clinical practice, the application of our findings calls for the implementation of comprehensive and early intervention strategies to address adverse outcomes of WPV. Firstly, in the initial phases of psychosocial events, healthcare institutions must respond promptly by implementing occupational health surveillance and workplace health promotion programs to facilitate early-stage recognition of WPV and mitigate severe burnout. Secondly, during and immediately following such events, families and healthcare institutions should provide timely support, focusing on relaxation techniques and conducive work environments to enhance the well-being of HWs, including the sleep hygiene and work ability enhancement. Lastly, long-term occupational care strategies should be instituted to foster and enhance individuals’ resilience.

In terms of health policy, our findings highlight the need for broader initiatives that address the systemic issues contributing to WPV and its downstream effects. Policymakers should consider regulations that protect HWs from WPV and provide resources for organizations to implement evidence-based interventions. Furthermore, the development of guidelines that emphasize the importance of sleep and work ability in the context of occupational health could help to standardize care and improve outcomes for HWs.

While single mediation pathways through sleep disturbance (accounting for 14.13%) and work ability (accounting for 31.84%) showed stronger effects than the chain mediation (accounting for 4.48%), this does not diminish the value of a comprehensive strategy. For hospitals with limited resources, prioritizing interventions that target sleep disturbance or work ability may be a pragmatic initial step. However, the ultimate goal should be to develop and implement a holistic program that addresses all aspects of this complex relationship.

### 4.5. Limitations

Some limitations still exist in our study. Firstly, the recruitment of HWs was conducted only from one secondary grade A comprehensive hospital in Wuhan, which may affect the representativeness of the data and lead to some sampling bias in this survey. Future research should aim to investigate HWs from diverse levels of hospitals by multicenter random sampling across different regions for a more comprehensive understanding. Secondly, given the cross-sectional design of this study, it is not conducive to confirming a causal relationship between WPV, sleep disturbance, work ability, and burnout. Therefore, further work should prioritize longitudinal or experimental studies to elucidate the causal relationships among these variables and explore other variables that may influence burnout. Thirdly, all variables were assessed using self-reported measures in this study. This may introduce response bias and social desirability bias.

## 5. Conclusions

In conclusion, this study established a chain mediating model to examine the impact of WPV on burnout among Chinese HWs two years after the COVID-19 outbreak, mediated by sleep disturbance and work ability. Specifically, this research provides valuable empirical insights for designing interventions to mitigate the adverse effects of psychosocial factors such as WPV and burnout. After exposure to WPV, intervention focused on reducing sleep disturbance and enhancing work ability among HWs may prove effective in alleviating burnout in subsequent interventions.

## Figures and Tables

**Table 1:** The characteristics of participants and distribution of the burnout score (*N* = 571).

Variable	Category	*n* (%)	Burnout		
			Mean ± SD	*t/F*	*P*
Gender	Female	448 (78.46)	5.07 ± 3.12	*t* = −0.012	0.990
	Male	123 (21.54)	5.07 ± 3.21		
Age (years)	<26	37 (6.48)	4.76 ± 2.93	*F* = 4.118	0.043 *
	26~	231 (40.46)	5.27 ± 3.31		
	36~	203 (35.55)	5.41 ± 3.17		
	46~	100 (17.51)	4.03 ± 2.44		
Department	General medicine	158 (27.67)	5.30 ± 3.10	*F* = 0.257	0.613
	Surgery	139 (24.34)	4.80 ± 2.84		
	Technical support	87 (15.24)	4.80 ± 3.32		
	Outpatient and emergency	86 (15.06)	5.90 ± 3.17		
	Administration department	101 (17.69)	4.61 ± 3.26		
Occupation	Doctor	163 (28.55)	5.62 ± 3.04	*F* = 9.436	0.002 **
	Nurse	275 (48.16)	5.01 ± 3.11		
	Technical support and administrative staff	133 (23.29)	4.51 ± 3.21		
Years of working	<1	7 (1.23)	2.86 ± 2.48	*F* = 1.184	0.277
	1~	85 (14.89)	4.93 ± 3.20		
	6~	131 (22.94)	5.37 ± 3.36		
	11~	131 (22.94)	5.39 ± 3.00		
	15~	91 (15.94)	5.51 ± 3.12		
	20~	126 (22.07)	4.33 ± 2.89		
Number of employees coworking	None/work alone	10 (1.75)	6.80 ± 3.08	*F* = 4.596	0.033 *
	<6	171 (29.95)	5.33 ± 3.25		
	6~	87 (15.24)	5.15 ± 3.51		
	11~	94 (16.46)	4.96 ± 3.32		
	16~	209 (36.60)	4.79 ± 2.74		
Shift work	Yes	436 (76.36)	5.22 ± 3.13	*t* = −2.072	0.039
	No	135 (23.64)	4.59 ± 3.11		
Night work	Yes	391 (68.48)	5.35 ± 3.14	*t* = −3.152	0.002
	No	180 (31.52)	4.47 ± 3.05		
Direct contact with patients	Yes	498 (87.22)	5.24 ± 3.11	*t* = −3.435	<0.001 ***
	No	73 (12.78)	3.92 ± 3.06		

Note: * *p* < 0.05; ** *p* < 0.01; *** *p* < 0.001.

**Table 2:** Means, standard deviations, and Spearman correlations between WPV, sleep disturbance, work ability, and burnout (*N* = 571).

	M	SD	1	2	3	4
1	5.07	3.13	1.000			
2	0.87	1.66	0.256 ***	1.000		
3	1.02	0.95	0.322 ***	0.208 ***	1.000	
4	34.70	6.08	−0.454 ***	−0.230 ***	−0.240 ***	1.000

Note: 1 = burnout, 2 = workplace violence, 3 = sleep disturbance, and 4 = work ability. *** *p* < 0.001 (two-tailed).

**Table 3:** Comparison of indirect effects of WPV on burnout mediated by sleep disturbance and work ability.

Pathways		Product of Coefficients		Bootstrapping BC 95% CIs	Percentage of Total Effect (%)
		β	Boot SE		
Effect					
Total effect		0.446	0.077	0.300, 0.602	100.00
Direct effect		0.220	0.072	0.069, 0.392	49.33
Indirect effect	Total indirect effects	0.226	0.048	0.133, 0.321	50.67
	X→M1→Y	0.063	0.020	0.027, 0.105	14.13
	X→M2→Y	0.142	0.041	0.065, 0.225	31.84
	X→M1→M2→Y	0.020	0.007	0.008, 0.036	4.48
Contrasts					
Model 1 versus Model 2		−0.079	−0.021	−0.038	—
Model 1 versus Model 3		0.043	0.013	0.019	—
Model 2 versus Model 3		0.122	0.034	0.057	—

Note: X = workplace violence; M1 = sleep disturbance; M2 = work ability; Y = burnout. Model 1 = workplace violence—sleep disturbance—burnout; Model 2 = workplace violence—work ability—burnout; Model 3 = workplace violence—sleep disturbance—work ability—burnout.

## Data Availability

The dataset is available on request from the corresponding author.
